# 

**Published:** 1859-12

**Authors:** 


### Contents of the Chicago Medical Journal--December, 1859.

### ORIGINAL COMMUNICATIONS.

PAGE.

ART. I.—Cases of Extirpation of the Parotid Gland and other Glandular Tumors. By Daniel Brainard, M.D., Professor of Surgery in Rush Medical College, Surgeon of the U. S. Marine Hospital, of the City Hospital, Chicago, etc. (Illustrated).... 709

ART. II.—Essay on Pneumonia. (Read before the Hendricks County Medical Society.) By A. Heavenridge, M. D., of Stilesville, Ind........ 718

ART. III.—A Case of Severe Burn treated by White Paint. Recovery, etc. By O. C. Gibbs, M.D., Frewsburg, Chautauque County, New York........ 730

ART. IV.—Report of Cases in Ophthalmic Practice. By E. L. Holmes, M.D., of Chicago. (Continued from page 652.) Rupture of both Eyes by "Gouging,"........ 733

### SELECTIONS.

Diseases of the Corpuscles of Blood. (From the *Medical News and Library*.)........ 738

The Structure and Functions of the Brain. (From the *Cleveland Medical Gazette*.).... 744

Transactions of the New Hampshire Medical Society, 1859.

- Amputation of Shoulder Joint, by Josiah Crosby, M.D........ 747
- Perforation of the Femur, by Josiah Crosby, M.D........ 747

### TRANSLATIONS.

On Inflammation of the Cervical Lymphatic Ganglia. (*Adenite Cervicale*.) By H. Larrey, Member of the Council of Health of the Army, etc........ 749

### PROCEEDINGS OF MEDICAL SOCIETIES.

Brownsburgh Medical Society........ 750

### MISCELLANEOUS MEDICAL INTELLIGENCE.

Aneurism of the Right Carotid and Subclavian Arteries; Ligation of the Arterial Innominata. By E. S. Cooper, M.D., San Francisco........ 751

Professor Paul F. Eve........ 752

The Chicago Charitable Eye and Ear Infirmary........ 754

Illinois State Medical Society........ 755

The Smethurst Case........ 757

### BOOK AND PAMPHLET NOTICES.

A System of Surgery—Pathological, Diagnostic, Therapeutic and Operative. By Samuel Gross, M.D., Professor of Surgery in Jefferson Medical College, etc. etc. Illustrated by 936 Engravings. In two vols. Pp. 1162 and 1196. Philadelphia: Lea & Blanchard. 1859........ 760

### EDITORIAL.

Woorara as a Remedy for Traumatic Tetanus........ 761

Surgical Items:

1. Reductions of Dislocations of the Hip Joint by Manipulation........ 762
2. Exsection of five Tarsal Bones for Caries........ 763
3. Strangulated Femoral Hernia........ 763
4. Amputation at the Hip Joint........ 764
5. Resection of the Head of the Humerus for Gunshot Wound........ 764

College of Pharmacy........ 765

Rush Medical College........ 765

The "Journal"........ 765

Prospectus of the Chicago Medical Journal for 1860........ 766

